# *In vivo* Analysis of Normal Optic Nerve in an Elderly Population Using Diffusion Magnetic Resonance Imaging Tractography

**DOI:** 10.3389/fneur.2021.680488

**Published:** 2021-09-24

**Authors:** Yeji Moon, Jin-Ju Yang, Won June Lee, Ji Young Lee, Yu Jeong Kim, Han Woong Lim, The Alzheimer's Disease Neuroimaging Initiative (ADNI), Michael W Weiner

**Affiliations:** ^1^Department of Ophthalmology, Seoul National University Hospital, Seoul, South Korea; ^2^Department of Ophthalmology, Hanyang University Hospital, Hanyang University College of Medicine, Seoul, South Korea; ^3^Hanyang Vision Research Center, Hanyang University, Seoul, South Korea; ^4^Department of Radiology, Hanyang University Hospital, Hanyang University College of Medicine, Seoul, South Korea

**Keywords:** diffusion magnetic resonance imaging, tractography, fractional anisotropy, mean diffusivity, optic nerve

## Abstract

**Purpose:** To quantitatively investigate the microstructural properties of the optic nerve (ON) *in vivo* using diffusion magnetic resonance imaging (dMRI) tractography in an elderly population and to determine the differences between the ON diffusion properties stratified by basic demographics.

**Methods:** We measured fractional anisotropy (FA), mean diffusivity (MD), radial diffusivity (RD), and axial diffusivity (AD) of the intraorbital ON in cognitively normal controls selected from the Alzheimer's Disease Neuroimaging Initiative 3 database (*n* =104; mean age = 73. 8 ± 8.1 years) using dMRI probabilistic tractography and evaluated the correlation between diffusion parameters and demographic factors. Diffusion parameters were measured in 20 equidistant nodes along the tract, and the data from proximal 70% (14 nodes) of the intraorbital ON were averaged.

**Results:** The mean FA of the intraorbital ON was 0.392 ± 0.063, and the mean MD was 1.163 ± 0.165 μm^2^/s. The mean RD was 0.882 ± 0.152 μm^2^/s, and the mean AD was 1.693 ± 0.183 μm^2^/s. The multiple linear regression model showed a negative correlation between FA and age. FA in females was significantly higher than males, whereas RD in female was significantly lower.

**Conclusions:** We measured the diffusion properties of the intraorbital ON using dMRI tractography in an elderly cognitively normal population. The diffusion properties detected by dMRI tractography may substantially reflect the microstructure of the ON.

## Introduction

The optic nerve (ON), the convergence of retinal ganglion cell axons, is considered as a part of the central nervous system, and it has been studied as a surrogate for various neurodegenerative diseases (1, 2). Optical coherence tomography (OCT), one of the most commonly used imaging techniques in ophthalmology, can provide *in vivo* images of the retina and ON with high resolution (<10 μm) (3, 4). However, it has several limitations, and the most significant is the penetration depth. OCT can hardly show deep structures beyond the sclera, such as the retrobulbar ON (5, 6).

Meanwhile, magnetic resonance imaging (MRI) shows structural changes throughout the ON, and diffusion MRI (dMRI) is sensitive to microstructural changes in the white matter. dMRI creates images based on the relative diffusion of water molecules in the tissue. Recently, dMRI has emerged as a useful tool to investigate the structure of nervous tissues at a microscopic level, although the resolution of images is a millimetric scale. It has the potential to provide information of the cellular organization of nervous tissues non-invasively and *in vivo*. Moreover, dMRI has been used to monitor the dynamic changes during neuronal activation, which suggests that it could perform as a functional neuroimaging modality (7).

**Figure 1:**
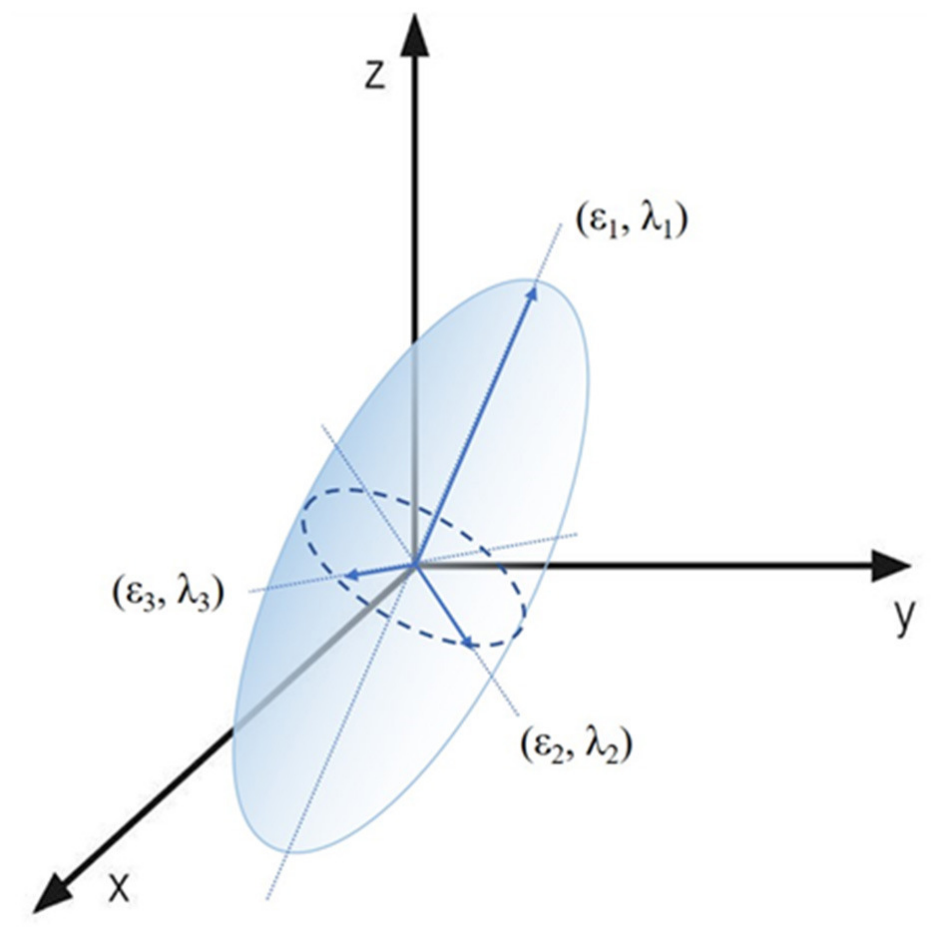
Visual presentation of the diffusion tensor model. The ellipsoid area of a water molecule diffusion can be characterized by three eigenvectors (ε_1_, ε_2_, ε_3_) and three eigenvalues (λ_1_, λ_2_, λ_3_).

Based on dMRI, diffusion tensor imaging (DTI) investigates the fiber architectures of brain white matter. The white matter and cranial nerves including the ON consist of bundles of axons. The axon membranes and myelin sheaths mainly induce diffusion restriction of water molecules. The direction of maximum diffusivity becomes parallel to the axonal direction, making the diffusion anisotropic (8, 9). Under the anisotropic condition, the probable location of a molecule after a certain time will be within an ellipsoid. DTI techniques were acquired using this ellipsoid mathematical model. The ellipsoid can be characterized by three vectors (ε_1_, ε_2_, ε_3_) called the eigenvectors, with corresponding lengths (λ_1_, λ_2_, λ_3_), the eigenvalues (Figure 1).

The most commonly used diffusion parameters in DTI are fractional anisotropy (FA) and mean diffusivity (MD) (10–12). FA is the normalized standard deviation (SD) of the three eigenvalues. Its value characterizes the fraction of molecular displacements ranging from 0 for completely isotropic diffusion to 1 for completely anisotropic diffusion. FA represents the directionality of diffusion, independently of the rate of diffusion. Although FA is highly sensitive to microstructural changes, it is not specific to each type of microstructural changes. Therefore, it is desirable to include other DTI measurements in any analysis. MD is the mean of three eigenvalues, and it is a measure of the ellipsoid volume as the overall mean displacement of water molecules. Thus, it reflects the mean amount of diffusion, irrespective of the direction of diffusion. MD is sensitive to cellularity, edema, and necrosis.

The apparent diffusivity is also measured as radial diffusivity (RD) or axial diffusivity (AD). RD is the apparent water diffusion in the direction perpendicular to the axonal fibers, whereas AD refers to the diffusivity along the main axis, which is defined as the magnitude of water diffusion parallel to the axonal fibers. RD and AD represent the eigenvalue amplitudes and demonstrate more specific relationships to pathologic changes (13). RD has been related to the degree of myelination, although axonal diameters or density may also influence RD. In contrast to RD, AD has been assumed to represent the axonal integrity (14, 15).

The three-dimensional reconstruction of nerve fiber tracts of DTI data is called tractography. The global architectures of fiber bundles are reconstructed by connecting the local information in voxels. The tract begins from a seed point and traced along the most likely direction. DTI studies using this technique have shown the change of diffusion properties in several eye diseases such as glaucoma and amblyopia (16–18). However, for the standard implementation of dMRI for ON evaluation, it is required to investigate the normal values of these parameters. It is particularly essential to assess the microstructural properties of ON and to determine reference values of diffusion parameters in detail for an aged population who are susceptible to neurodegenerative changes. Aging process including axonal disintegration and demyelination can change the diffusion properties of ON in elderly population.

Therefore, in this study, we aimed to quantitatively investigate *in vivo* ON diffusion properties using dMRI tractography and determine the change of ON diffusion properties according to age and sex in an elderly population using the data from Alzheimer's Disease Neuroimaging Initiative (ADNI) 3. Although ADNI is a consortium established to develop standardized imaging techniques for Alzheimer disease, and the database from ADNI is focused on the brain, not ON, it gives us a standardized large-scale neuroimaging data with a wide range of age.

## Materials and Methods

### Subjects

The MRI data used in this observational study were obtained from the database of the third phase of the ADNI (ADNI3; http://adni.loni.usc.edu). The ADNI3 data were collected for more than 300 participants using new dMRI protocols for all GE, Siemens, and Philips scanners at 47 sites across the United States and Canada. ADNI data are publicly available, and they have been used in various studies (19, 20). ADNI research activities were reviewed and approved by the institutional review board (IRB) of each participating facility, and written informed consent was obtained from each participant. This study was exempted from IRB approval by the IRB of Hanyang University Hospital because it involved a secondary analysis of deidentified ADNI data. This study followed the tenets of the Declaration of Helsinki for biomedical research.

Each subject was scanned during two or more visits, separated by at least 6 months, and ADNI3 is still ongoing. We selected normal controls who underwent only Siemens 3 Tesla MRI (Basic Skyra E11 and Prisma D13; Siemens, Erlangen, Germany) to collect the largest cohort of normal patients with a wide age range and avoid different scan acquisition parameters, including vendor, voxel size, and angular resolution, known as the scanner effect on diffusion measures (21–24). Of 144 normal controls at the baseline visit with ages ranging from 55 to 96 years, one subject was excluded because he showed high signal intensity for his left eye on T1-weighted MRI scans, and 39 subjects were excluded because of failure of image processing; 104 of the final sample (mean age with SD: 73.8 ± 8.1 years; 39/65, male/female) were included.

### MRI Scan Acquisition

As mentioned previously, all subjects underwent Siemens 3 Tesla MRI (Basic Skyra E11 and Prisma D13; Siemens). T1-weighted anatomical images were obtained using an MPRAGE sequence with a 1 × 1 × 1-mm voxel size and a 2,300-ms repetition time (TR). For diffusion-weighted MRI, the pulse sequence was acquired in the axial plane (56-ms echo time; 7,200-ms TR; two *b* values with *b* = 0 and *b* = 1,000 s/mm^2^; 48 diffusion directions, 7 non–diffusion-weighted images, 2 × 2 × 2-mm voxel size). Further details on scan parameters can be found at https://adni.loni.usc.edu/wp-content/uploads/2017/07/ADNI3-MRI-protocols.pdf.

### Image Preprocessing

DTI data were preprocessed for denoising and bias field correction using MRtrix3 software package (http://www.mrtrix.org) based on previously published methods (25–29). Subsequent processing was performed using the mrDiffusion software package (https://github.com/vistalab/vistasoft). The anatomical T1-weighed images were aligned to the anterior commissure–posterior commissure plane. Diffusion-weighted images were corrected for eddy currents and subject motions (30). The motion-corrected non–diffusion-weighted (b0) images were averaged and aligned to the structural T1-weighted images using a rigid body mutual information algorithm. Diffusion-weighted gradient directions were reoriented by applying the same transformation used on the diffusion-weighted images. All images from the diffusion sequence were resampled to 2-mm isotropic voxels using a trilinear interpolation algorithm. Finally, the tensors were optimized for estimation using a least-squares algorithm with bootstrapping for 500 times. The resulting eigenvalues were used to compute FA, MD, RD, and AD (31). In this procedure, all images were thoroughly reviewed by a single author (J-J.Y.), and the images with failed processing were excluded.

### Regions of Interest for Fiber Tractography

**Figure 2:**
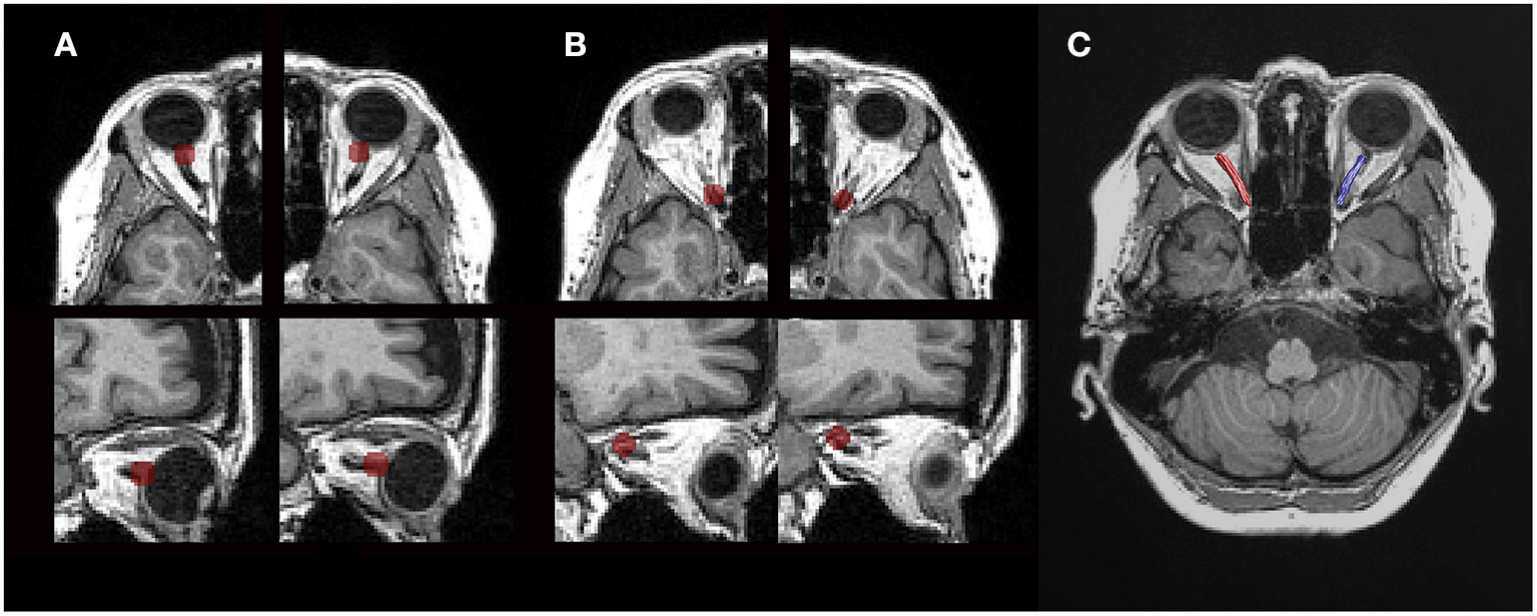
Tractography using diffusion magnetic resonance imaging. **(A)** Axial and sagittal views (top to bottom) of the regions of interest (ROIs). A 4-mm-radius spherical ROI just posterior to the globe was centered on the emerging optic nerve in each eye. **(B)** Another 4-mm-radius spherical ROI was within the orbital apex, and its center was placed in the center of the annulus of Zinn. **(C)** Cleaned tractography-generated optic nerve pathways. Visualization of the ROIs and tractography was generated with the MATLAB Brain Anatomy software package (https://github.com/francopestilli/mba).

We manually identified two ROIs for each intraorbital ON. To identify the starting point for the ON, we placed a 4-mm-radius spherical ROI just posterior to the globe of each eye and centered on the emerging ON (Figure 2A). Another 4-mm-radius spherical ROI was placed in the orbital apex, and its center was placed in the center of the annulus of Zinn, where the extraocular muscles were confluent (Figure 2B). The placement of ROIs was based on the gross anatomical landmarks in the T1-weighted and b0 images. We reviewed the images thoroughly, and when there was a difference between the locations of the ROIs of the T1-weighted and b0 images, we choose b0 images to locate the ROI. The diameter of the ROIs was larger than those of the ONs of the participants to prevent undersampling at the tractography stage.

### Fiber Tracking and Cleaning

The ON was identified using probabilistic fiber tractography to calculate the most likely pathway between a pair of ROIs using ConTrack (https://github.com/vistalab/contrack). This tool was used to identify visual pathways in previous studies (32, 33). The most likely pathway was generated from a set of 1,000 candidate pathways between the ON head and orbital apex. The ConTrack scoring algorithm was used to select the 100 most likely fibers (top 10%).

A fiber was automatically removed when its distance was more than 2.4 SDs away from the pathway's fiber core or more than 3 SDs longer than the average fiber length, following the prescriptions of a previous study (17). Fiber cleaning was performed using the Automated Fiber Quantification (AFQ) toolkit (https://github.com/jyeatman/AFQ). The pathways of all participants were processed using the same AFQ cleaning parameters. After automated cleaning, all the ON pathways were subjected to quality assessment, and additional manual cleaning was applied as necessary by a single author (Y.M.) (Figure 2C).

### Diffusion Measures Based on Tractography

Although one diffusion parameter may be highly correlated with a certain type of microstructural alterations, it seems necessary to consider various diffusion parameters together to differentiate the pathologic changes. Adding RD and AD helps understanding which component of the diffusion tensor contributes to changes of MD and FA. For instance, FA can decrease as a result of an increase in RD or a decrease in AD. Conversely, we would not know whether the change of AD or RD is significant enough to affect the anisotropy or the overall diffusivity by measuring only AD and RD. It is advantageous to consider all four parameters when investigating the diffusion properties of nervous fibers.

The following formulas were used for each diffusion parameter:


$$\begin{array}{l} \;FA=\;\sqrt{\frac{{\left(\lambda_{1}-\lambda_{2}\right)}^{2}+{\left(\lambda_{2}-\lambda_{3}\right)}^{2}+{\left(\lambda_{3}-\lambda_{1}\right)}^{2}}{2({\left(\lambda_{1}\right)}^{2}+{\left(\lambda_{2}\right)}^{2}+{\left(\lambda_{3}\right)}^{2})}}\\ MD=\;\frac{\left(\lambda_{1}+\lambda_{2}+\lambda_{3}\right)}{3}\\ \;RD=\;\frac{\left(\lambda_{2}+\lambda_{3}\right)}{2}\\ \;AD=\;\lambda_{1} \end{array}$$

We measured the diffusion properties in 20 equidistant nodes along the ON to normalize the pathway length and facilitate statistical analysis. The values for diffusion properties were generated for each node using a Gaussian-weighted average, where the central tract was weighted over the outlying fibers. This approach has been described in detail in previous studies (16, 17, 34). Finally, to mitigate the effect of eye movement during MRI acquisition, diffusion parameters from posterior 70% (14 nodes) of intraorbital ON were included and averaged in final analysis.

### Statistical Analysis

A strong correlation between right and left eye diffusion parameters was observed (FA, Pearson *r* = 0.799, *p* < 0.001; MD, Pearson *r* = 0.740, *p* < 0.001; RD, Pearson *r* = 0.725, *p* < 0.001; AD, Pearson *r* = 0.625, *p* < 0.001), and the analyses in this study were performed using average values from both eyes. A two-tailed *p* ≤ 0.05 was considered statistically significant.

The Pearson correlation coefficient was used to determine the correlation between diffusion measures and age. A two-sample *t*-test was used to test for sex-related differences in diffusion measures. In this study population, the female participants were significantly younger than the male participants (male vs. female, 75.9 ± 8.6 vs. 72.5 ± 7.6 years, *p* = 0.038). Therefore, we performed multiple linear regression analysis using both age and sex to adjust for potentially confounding effects.

### Data Availability Policy

The data that support the findings of this study are available from the study team upon reasonable request.

## Results

The mean FA of the intraorbital ON was 0.392 ± 0.063, and the mean MD was 1.163 ± 0.165 μm^2^/s. The mean RD was 0.882 ± 0.152 μm^2^/s, and the mean AD was 1.693 ± 0.183 μm^2^/s.

FA of the intraorbital ON was negatively correlated with age, whereas RD showed a positive correlation (FA with age, Pearson *r* = −0.338, *p* < 0.001; RD with age, Pearson *r* = 0.211, *p* = 0.031). Meanwhile, MD and AD had no significant correlation with age (MD with age, Pearson *r* = 0.137, *p* = 0.167; AD with age, Pearson *r* = −0.028, *p* = 0.778).

The mean FA and RD of ON between the male and female subjects were significantly different; female subjects had higher FA and lower RD (FA in male vs. female, 0.360 ± 0.052 vs. 0.411 ± 0.062, *p* < 0.001; RD in male vs. female, 0.964 ± 0.161 vs. 0.875 ± 0.171 μm^2^/s, *p* = 0.003). The differences in the mean MD and AD between male and female subjects were not statistically significant (MD in male vs. female, 1.200 ± 0.167 vs. 1.140 ± 0.161 μm^2^/s, *p* = 0.072; AD in male vs. female, 1.687 ± 0.186 vs. 1.697 ± 0.183 μm^2^/s, *p* = 0.795).

For FA and RD that were correlated with both age and sex in univariable analysis, we performed multiple linear regression. Finally, FA showed significant correlation with both age and sex, whereas RD had significant correlation with only sex (Table 1).

**Table 1:** Multiple linear regression model for fractional anisotropy and radial diffusivity of the optic nerve.

**Diffusion parameter**	**Age**		**Sex^a^**		**Adjusted** ***R***^**2**^
	**Standardized coefficient**	***p*** **-value**	**Standardized coefficient**	***p*** **-value**	
Fractional anisotropy	−0.269	**0.003**	0.337	**<0.001**	0.207
Radial diffusivity	N/A	0.079	−0.221	**0.024**	0.040

a *The reference group is male. The numbers in bold font indicate statistically significant correlations*.

## Discussion

In this study, we determined the normal values of parameters of the intraorbital ON using dMRI probabilistic tractography in an elderly population. We found that FA had a negative correlation with age. Females had higher FA and lower RD than males.

In previous studies using tractography for ON analysis in various diseases, the ROIs were placed at the optic chiasm as the ending point (16, 17). In contrast to these studies, we analyzed the diffusion parameters of only the intraorbital parts of ON. When tractography, involving the intracanalicular and intracranial ON, was performed, the diffusivity along the ON fluctuated. This implies that the extracted tractography included heterogeneous tract fibers that were not confined to the ON. Compact tissues around the ON in the optic canal and the cranial cavity may make false tracts of the visual pathway. Moreover, the placement of ROIs is important because it can induce significantly different outcomes of the final tractography. In the ADNI3 database, diffusion sequences had image distortions; diffusion scans acquired with opposite phase-encoding directions were not used. As mentioned in section Materials and Methods, we prioritized b0 MRI scans when the anatomical structures in the T1-weighted and b0 MRI scans were not completely matched, and the optic chiasm in b0 MRI scans could not be located in several cases. To reduce errors for ROI placement and perform more accurate tractography, we placed ROIs in the center of the annulus of Zinn, which is a confluence of the extraocular muscles, to facilitate the identification of the ON pathways.

Age-related ON changes in various animals have been previously reported (35–40). In summary, age-related changes may be classified into four: (i) axonal changes, (ii) myelin abnormalities, (iii) changes in neuroglial cells, and (iv) connective tissue thickening. Aging axons undergo various degenerative changes, and the number of nerve fibers decreases with aging. Meanwhile, the volume of aging axons and the thickness of the entire ON fiber increase. Myelin sheaths also become thicker as the thickness of the axons increases. In addition, morphological alterations of myelin sheaths, including ballooning, widening of sheath lamellae, partial loss, and demyelination, have been observed. Microglia are commonly observed among degenerating axons, and the number of astrocytes also increases. They undergo hypertrophy, and their processes occupy space vacated by degenerated nerve fibers. Last, aging ONs demonstrate thickening of the trabeculae of connective tissue. In studies involving the human ON, similar changes have been reported (41–43).

In the current study, FA decreased with age. Age-related changes in the microstructure of the ON contribute to the decrease in FA detected by dMRI. The white matter and cranial nerves, including the ON, consist of bundles of axons, whose membranes and myelin sheaths mainly induce diffusion restriction of water molecules. The direction of maximum diffusivity becomes parallel to the axonal direction, making the diffusion anisotropic (8, 9). Because the parallel organization of nerve fiber bundles is the basis for diffusion anisotropy and myelin sheaths seem to modulate the amount of anisotropy (44), the degeneration of axon and myelin sheaths may reduce the anisotropy of the diffusion process.

Interestingly, MD showed no significant correlation with aging in the multiple regression model. RD showed positive correlation with aging with borderline significance. The changes in the amount of diffusivity have been various in previous studies for the age-related changes of the white matter in multiple regions, although they have consistently reported a decrease in FA (12). Because AD tends to be more variable according to pathologic changes, age-related decreases as well as increases in AD have been reported, resulting in no net difference, increase, or decrease in MD. In contrast to AD, multiple studies suggest that RD changes are more prominent according to age (45–48), and most studies have reported that RD has decreased with increasing age. However, a few studies have revealed a decrease in AD and MD without a difference in RD (49, 50). Based on these different patterns of diffusivity changes, several studies have classified the regions of the brain according to the specific age-related pathologic change. When applied to this classification, the results from the ON in this study, a decrease in FA accompanied with increase in RD, suggest that the main pathologic changes in the ON aging process are the subtle microstructural alterations with predominant demyelination, which is like the forceps major (posterior thalamic radiations/optic radiations) and superior corona radiata. Further studies are required to answer the question of why the aging process occurs in various ways according to region of the nervous system.

In the meantime, Sun et al. (51) previously reported that there was no obvious age-related difference in diffusion parameters of ON and optic radiation in DTI. In that study, the authors measured the diffusion parameters in ROI at the level of the middle part of intraorbital ON. In addition, they included healthy subjects aged from 18 to 62 years (mean age = 40.3 ± 12.5 years) who were younger than our study subjects. Therefore, it should be noted that diffusion properties of the ON before the sixth decade of life may not show any significant age-related change.

In the present study, females had higher FA and lower RD than males, which is consistent with the reports by other studies on sex-related differences in ON structure and function. Various studies have previously reported the association of the male sex with thinner retinal nerve fiber layers on OCT (52–55), whereas the optic disc area is larger in males than in females (56–58). In addition to structural differences, several differences in visual evoked potentials have been revealed, such as shorter latency and high amplitude in females (59, 60). Considering that we analyzed the data from an elderly population, it is highly advisable to consider that sex-related difference could become more prominent due to the difference in the course of ON neurodegeneration between females and males in this study population. Retinal gene expression responses to aging have been reported to be sexually divergent (61). Therefore, sex-related difference in diffusion properties of ON should be sufficiently considered in an elderly population.

**Figure 3:**
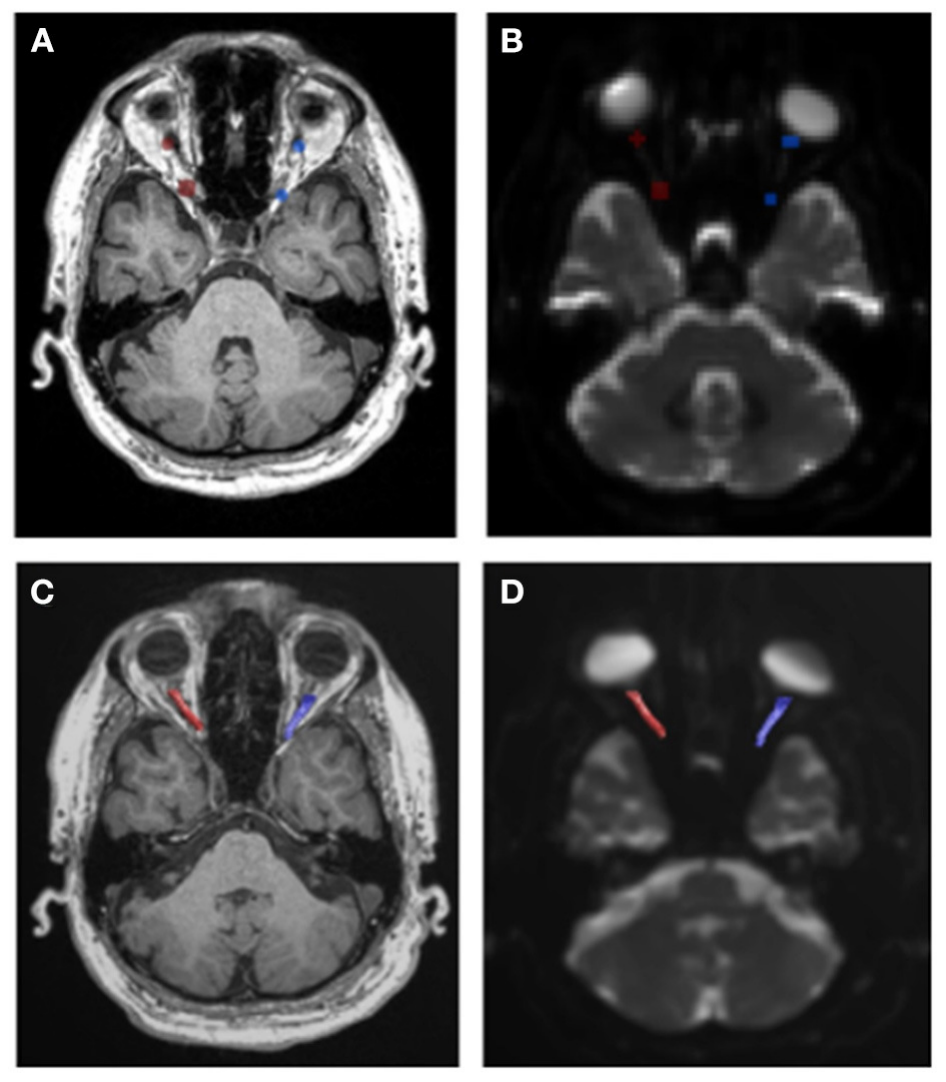
Geometric distortion in diffusion magnetic resonance images. **(A,B)** When there was a difference between the locations of the ROIs of the T1-weighted image and b0 image, we choose b0 images to locate the ROI. **(C,D)** Final tractography is well-matched with the b0 image rather than the T1-weighted image.

This study has several limitations that should be addressed. First, the dMRI data used in this study were obtained from the ADNI3, and all the subjects with normal cognitive functions in ADNI3 may not have been ophthalmologically normal because of the limited ophthalmologic information of the participants. However, all of them had adequate visual acuity to allow neuropsychological testing according to the inclusion criteria of ADNI. In addition, we used average values from right and left ON measurements and not measurements from only one eye, to reduce the error induced by including eyes with diseases. Second, the data from ADNI3 are not intended for the analysis of ON. Accordingly, eye movement correction was not applied. To alleviate the artifacts induced by the eye movement, we only used the data from the proximal 70% of ON. Third, it should also be noted that the voxel size was 2 × 2 × 2 mm. Given that the width of intraorbital ON is ~3 mm, the results could be affected by partial volume effects. Last, in addition to the image distortion caused by a single phase-encoding direction, a mismatch between the T1-weighted image and b0 MRI scan was induced. By prioritizing b0 MRI scans when placing the ROIs, we attempted to overcome this limitation (Figure 3). Furthermore, we averaged the diffusion parameters from each node of ON to mitigate this geographic distortion.

Despite these limitations, to the best of our knowledge, this is the first study to investigate the diffusion properties in normal ON using well-established large-scale, multisite neuroimaging data that match T1-weighted imaging and DTI. As the ADNI3 data have a wide range of age distribution, it was possible to analyze the effect of age on diffusion properties of ON. Because it is open data, it is also possible to verify the reproducibility of our results.

This study showed the diffusion properties of the ON stratified by age and sex. Young age was correlated with higher FA, and females had higher FA and lower RD than males. Therefore, the differences based on age and sex should be considered when evaluating the ON using dMRI. Moreover, the results from the present analysis correspond well with those of previous studies on the ON structures and functions. This implies that the diffusion parameters along the ON determined using dMRI tractography may substantially reflect the ON microstructure and play roles as surrogates for various optic neuropathies. dMRI can help improve the understanding of the pathophysiology of multiple optic neuropathies and provide useful information for the diagnosis and prognostic prediction of various ON diseases.

## Data Availability Statement

The raw data supporting the conclusions of this article will be made available by the authors, without undue reservation.

## Ethics Statement

The ADNI research activities were reviewed and approved by the Institutional Review Board (IRBs) of each participating facility. The patients/participants provided their written informed consent to participate in this study.

## Author Contributions

WL, YK, and HL contributed to conception and design of the study. J-JY and JL contributed to acquisition of data. YM and J-JY analyzed and interpreted the data. YM wrote the first draft of the manuscript. J-JY wrote sections of the manuscript drafted and revised the manuscript for intellectual content. All authors contributed to manuscript revision, read, and approved the submitted version.

## Funding

This research was supported by the Basic Science Research Program of the National Research Foundation of Korea (NRF). Korean government funding was received from the Ministry of Science, ICT and Future Planning (MSIT) (No. NRF-2019R1A2C4070638, to HL) and from the Bio & Medical Technology Development Program of the NRF (No. NRF-2019M3E5D1A01069352, to WL).

## Conflict of Interest

The authors declare that the research was conducted in the absence of any commercial or financial relationships that could be construed as a potential conflict of interest.

## Publisher's Note

All claims expressed in this article are solely those of the authors and do not necessarily represent those of their affiliated organizations, or those of the publisher, the editors and the reviewers. Any product that may be evaluated in this article, or claim that may be made by its manufacturer, is not guaranteed or endorsed by the publisher.

## List of Co-investigators From Alzheimer's Disease Neuroimaging Initiative

Michael W Weiner, (UCSF, NCIRE, VA, Principal Investigator); Paul Aisen, (University of California, San Diego, PI of Clinical Core); Ronald Petersen, (Mayo Clinic, PI of Clinical Core); Clifford Jack, (Mayo Clinic, Rochester, Minnesota, PI of MRI Core); Matthew Bernstein, (Mayo Clinic, Rochester, MRI Core Leaders and Key Personnel); Nick Fox, (University of London, MRI Core Leaders and Key Personnel); Paul Thompson, (UCLA School of Medicine, MRI Core Leaders and Key Personnel); Norbert Schuff, (UCSF MRI, MRI Core Leaders and Key Personnel); Charles DeCArli, (UC Davis, MRI Core Leaders and Key Personnel); Bret Borowski, (Mayo Clinic, MRI Core Leaders and Key Personnel); Jeff Gunter, (Mayo Clinic, MRI Core Leaders and Key Personnel); Matt Senjem, (Mayo Clinic, MRI Core Leaders and Key Personnel); Prashanthi Vemuri, (Mayo Clinic, MRI Core Leaders and Key Personnel); David Jones, (Mayo Clinic, MRI Core Leaders and Key Personnel); Kejal Kantarci (Mayo Clinic, MRI Core Leaders and Key Personnel); Chad Ward (Mayo Clinic, MRI Core Leaders and Key Personnel); Laurel Beckett, (University of California, Davis, PI of Statistics Core).
